# Soluble Flt-1 in AMI Patients Serum Inhibits Angiogenesis of Endothelial Progenitor Cells by Suppressing Akt and Erk’s Activity

**DOI:** 10.3390/biology11081194

**Published:** 2022-08-09

**Authors:** Lijie Zhang, Xingkun Zhang, Xiaoming Zhong, Mengya Fan, Guoliang Wang, Wei Shi, Ran Xie, Yinxiang Wei, Hailong Zhang, Xiangxu Meng, Yaohui Wang, Yuanfang Ma

**Affiliations:** 1 Joint National Laboratory for Antibody Drug Engineering, The First Affiliated Hospital of Henan University, Henan University, Kaifeng 475004, China; 2 Henan Key Laboratory of Coronary Heart Disease Control & Prevention, Department of Cardiology, Central China Fuwai Hospital, Zhengzhou 450003, China; 3 Department of Cardiology, Henan Provincial People’s Hospital, Zhengzhou 451450, China; 4 Department of Cardiology, Huaihe Hospital of Henan University, Kaifeng 475000, China; 5 Department of Cardiovascular, the First Affiliated Hospital of Henan University, Kaifeng 475004, China

**Keywords:** acute myocardial infarction, endothelial progenitor cells, angiogenesis, sFlt-1

## Abstract

**Simple Summary:**

Acute myocardial infarction (AMI) is the leading cause of mortality in the world. Endothelial progenitor cells (EPCs) exert important roles in the recovery of collateral circulation via angiogenesis. In this study, we studied the characteristics of EPCs isolated from the peripheral blood of AMI patients and healthy subjects. We found that the number of EPCs increased in AMI patients and exhibited faster migration compared to healthy subjects. However, no difference in angiogenic activity was observed in EPCs between AMI patients and healthy subjects. Interestingly, the serum level of sFlt-1 was elevated in AMI patients. Further analysis demonstrated that sFlt-1 inhibited EPCs angiogenesis in vitro by inhibiting the Akt and Erk signaling pathways. In conclusion, our study uncovered that EPCs increased in quantity, but their angiogenesis activity was inhibited by serum sFlt-1 in AMI patients.

**Abstract:**

In acute myocardial infarction (AMI), endothelial progenitor cells (EPCs) are essential for the recovery of collateral circulation via angiogenesis. Clinical research has shown that the poor prognosis of the patients with AMI is closely associated with the cell quantity and function of EPCs. Whether there are differences in the biological features of EPCs from AMI patients and healthy subjects is worth exploring. In this study, EPCs were isolated from human peripheral blood and identified as late-stage EPCs by flow cytometry, immunofluorescence, and blood vessel formation assay. Compared to healthy subjects, AMI patients had more EPCs in the peripheral blood compared to healthy subjects. In addition, EPCs from AMI patients exhibited higher migration ability in the transwell assay compared to EPCs from healthy subjects. However, no difference in the angiogenesis of EPCs was observed between AMI patients and healthy subjects. Further studies revealed that soluble vascular endothelial growth factor receptor 1 (sFlt-1) in the serum of AMI patients was involved in the inhibition of EPCs angiogenesis by suppressing the Akt and Erk pathways. In conclusion, this study demonstrated that elevated serum sFlt-1 inhibits angiogenesis of EPC in AMI patients. Our findings uncover a pathogenic role of sFlt-1 in AMI.

## 1. Introduction

The predominant strategies for acute myocardial infarction (AMI) treatment in the clinic are drug-mediated thrombolysis or percutaneous coronary intervention (PCI), which recanalizes the clogged vessel, reducing infarction size [1]. However, the reperfusion can exacerbate myocardial cell death and vascular injury. Heart failure constitutes a serious complication of AMI [2,3]. It has been reported that the dysfunction of vascular endothelial cells is an initiating factor for the subsequent cardiovascular disease. On the other hand, the angiogenesis of endothelial cells plays a key role in the recovery from myocardial injury [4].

Endothelial progenitor cells (EPCs) are bone marrow-derived cells that differentiate into mature endothelial cells (ECs) and secrete proangiogenic factors [5,6,7]. In AMI patients, the number of EPCs in the peripheral blood is significantly increased [8]. EPCs act as a backup storehouse for ECs. When the ischemia occurs, EPCs differentiate into ECs participating in angiogenesis and neovascularization, and EPC-derived ECs in hypoxia or ischemia conditions account for more than 25% of ECs population [9,10]. However, the main difference between AMI-derived EPCs and EPCs from healthy subjects remains unclear.

Numerous studies have shown that angiogenesis mainly depends on vascular endothelial growth factor (VEGF), fibroblast growth factor, and angiogenin [11]. VEGF triggers angiogenesis by activating two receptors, VEGFR1 (Flt-1) and VEGFR2 (KDR). KDR is the major receptor to initiate the angiogenesis, while Flt-1 acts in the initial stages of vascular development [12]. Flt-1 has two isoforms due to alternative mRNA splicing, a soluble form (sFlt-1) and a membrane form (mFlt-1) [13], which exert opposite roles in angiogenesis. Soluble Flt-1 is antiangiogenic, and mFlt-1 is proangiogenic. The levels of sFlt-1 are upregulated in the serum of early onset of AMI and return to normal levels 8 h after AMI surgery [14]. Soluble Flt-1 is predominantly expressed by macrophages and ECs [13,15,16,17]. After binding with VEGF to form a heterodimer, sFlt-1 inhibits the VEGF-VEGFR2 signaling pathway, therefore, inhibits angiogenesis, which then impairs the recovery of patients.

In this study, we investigated the characteristics of EPCs isolated from the peripheral blood of AMI patients and healthy subjects. EPCs in the peripheral blood of AMI patients showed an increasing number and exhibited a high migration ability compared to those of healthy subjects. No difference was found in angiogenesis ability and sFlt-1/VEGF/KDR mRNA expression. Soluble Flt-1 inhibits the angiogenesis of EPCs via p-Akt and p-Erk signaling. Our findings uncover a pathogenic role of sFlt-1 in AMI. This study sheds light on a new strategy for the treatment of AMI with EPCs.

## 2. Materials and Methods

### 2.1. Patients and EPC Isolation

Peripheral blood mononuclear cells were collected from AMI patients at the Department of Cardiology, Huaihe Hospital of Henan University (Henan, China). The AMI patients (mean age, 64 years; 4 men and 3 women) were clinically diagnosed with coronary angiography, and blood samples were collected within 8 h after AMI onset. The blood samples from AMI patients were collected before cardiac angiography. Patient demographics and clinical characteristics are summarized in Table S1. Control subjects (mean age, 30 years; two men and four women) were healthy people without cardiovascular disease. All participants were informed of the purpose of the study and received a consent document. All study procedures followed the guidelines of the Declaration of Helsinki regarding the use of human blood and were approved by the ethics committee of the first affiliated hospital of Henan University (No. 2021-03-08).

EPCs in the peripheral blood were isolated using density gradient centrifugation. Briefly, peripheral blood mononuclear cells were isolated using density gradient centrifugation and then inoculated at 5 × 10^5^/cm into fibronectin-coated, six-well plates in endothelial basal medium 2 (EBM-2; HyClone, Logan City, UT, USA) containing 20% fetal bovine serum (FBS; GIBCO, Grand Island, NY, USA), vascular endothelial growth factor, insulin-like growth factor, ascorbic acid, heparin, and antibiotics. The cells were cultured in a 37 °C incubator with 5% CO_2_ for 4 days, and the nonadherent cells were removed by washing with PBS. The adherent cells were cultured for another 3 days before subsequent experiments. EPCs were confirmed by assessing the surface markers, such as CD34, CD133, and CD45 with flow cytometry analyses, and the same isotype antibodies were used as the negative controls.

### 2.2. Flow Cytometry Analysis of EPCs

The EPCs were identified by flow cytometry [18,19]. Cells were washed twice with cold PBS and incubated with rabbit PE-CD133 (Miltenyi Biotec, Bergisch Gladbach, Germany) and AF647-KDR (Becton Dickinson, Franklin Lakes, NJ, USA) for 30 min at room temperature. The normal rabbit IgG labeled by fluorescein was used as a control to define the negative population for each stain. CD31 (Becton Dickinson, Franklin Lakes, NJ, USA) and CD34 (Becton Dickinson, Franklin Lakes, NJ, USA) were not directly labeled and needed to be incubated with labeled secondary antibodies before flow cytometry. Cells were analyzed by Calibur (BD).

For the testing of EPC number, the CD34-positive population of mononuclear cells was isolated from the peripheral blood cell population, and then CD45dim cells were selected from the CD34-positive population. CD34/CD45dim cells were further sub-gated to identify the KDR-positive cell population, and the CD133-positive cell population was selected from the KDR-positive cell population.

### 2.3. Immunofluorescence Identification of EPCs

To detect the expression of vWF and KDR in EPCs, cells were grown on a glass dish with a diameter of 3.5 cm, fixed with 4% paraformaldehyde for 30 min, incubated with 0.1% Triton X-100 (Sigma-Aldrich, St. Louis, MO, USA) for 2 min, and blocked with 2% BSA in PBS for 60 min. Then, the cells were incubated with vWF (Merck Millipore, Billerica, MA, USA) and KDR antibodies overnight at 4 °C. The cells were washed with PBS, incubated with the corresponding fluorescence-labeled secondary antibody for 2 h, and stained with DAPI for 5 min at room temperature. Cells were next observed under a confocal microscope (Nikon, Tokyo, Japan).

FITC-UEA-I (Sigma-Aldrich, St. Louis, MO, USA), as a marker of human ECs, is used in combination with Dil-ac-LDL (Molecular Probes, Eugene, OR, USA) to label EPC differentiation. EPCs were incubated at a final concentration of 10 µg/mL Dil-ac-LDL for 4 h at 37 °C before fixation and 10 µg/mL FITC-UEA-I for 2 h at 37 °C.

### 2.4. Functional Identification of EPCs

Tube formation capacity was analyzed using a tube formation assay [20,21]. Matrigel (Corning, Tewksbury, MA, USA) was added into a 24 well plate and cultivated for 30 min in a cell incubator. EPCs were added to the 24-well culture plate at 8 × 10^4^ cells/well. After 6 h, the tubular structures were observed and counted.

The migration of EPCs was examined using the scratch assay. A total of 2 × 10^5^ cells were plated onto a six-well plate and were allowed to reach 80% confluence. The cells were starved by adding serum-free EGM-2 medium for 8 h. The cells were scratched with a 200 µL pipette tip, and then washed three times with PBS. Next, the medium containing 1% FBS was added to continue culturing. Cell migration was monitored every 4 h by optical microscopy.

EPC viability was measured using the MTS assay. EPCs were seeded at a density of 1 × 10^3^ cells per well in a 96-well plate with serum-free EGM-2 medium, and then cultured in a 5% CO_2_ incubator at 37 °C for 12 h. The cells were treated with DMEM containing 0.5% FBS in three replicate wells designed for different experimental groups, and then washed three times with PBS. Then, 10 μL of MTS reagent was pipetted into each well and incubated for 3.5 h. Absorbance at 490 nm was then surveyed by a microplate reader.

### 2.5. Real-Time Quantitative Polymerase Chain Reaction

RT-qPCR assays were performed for detecting the levels of VEGF, VEGFR2, sFlt-1, mFlt-1, and KDR genes. Total RNA obtained from EPCs was extracted as previously described and used to synthesize the cDNA [22]. RT-qPCR assays were finished with SYBR™ Select Master Mix (Thermo Fisher Scientific, Waltham, MA, USA) under the following conditions: denaturation at 95 °C for 10 s, followed by annealing and extension at 50 °C for 30 s, for a total of 40 cycles. The sequence of primers used in this study is presented in Table 1. The β-actin transcript was used as an endogenous reference to assess the relative level of mRNA transcript. The data were presented as relative fold change with respect to the control sample.

### 2.6. Western Blot

EPCs were washed three times using PBS and lysed on ice with a lysis buffer containing phosphatase inhibitor cocktail and a protease cocktail inhibitor. Protein levels in the lysate and supernatant were measured via BCA Protein Quantification Kit (Cwbiotech Company, Taizhou, China). Proteins were denatured with SDS sample buffer, boiled at 100 °C for 10 min, separated by SDS-PAGE gels, and transferred onto NC membranes. After blocking with TBST containing 5% BSA, membranes were incubated with VEGFR2, Akt, cleaved Akt, Erk1/2, and cleaved Erk1/2 antibodies overnight at 4 °C, respectively. Then, the membranes were incubated with the appropriate secondary antibodies coupled to HRP for 2 h at room temperature. The protein bands were detected with Pierce™ ECL Western Blotting Substrate and scanned by an automatic chemiluminescence imaging system (Tanon, Shanghai, China).

### 2.7. Enzyme-Linked Immunosorbent Assay

The concentrations of VEGF and sFlt-1 in serum from AMI patients and healthy subjects were measured using corresponding ELISA kits according to the manufacturer’s instructions (R&D Systems, Abingdon, UK) [23].

### 2.8. Statistical Analysis

All values were presented as the means ± SEM. Statistical significance was determined by the unpaired Student *t*-test for two groups and one-way analysis of variance (ANOVA) for multiple groups using GraphPad Prism 5.0 software (* *p* < 0.05, ** *p* < 0.01, and *** *p* < 0.001).

## 3. Results

### 3.1. Isolation, Culture, and Identification of EPCs

**Figure 1:**
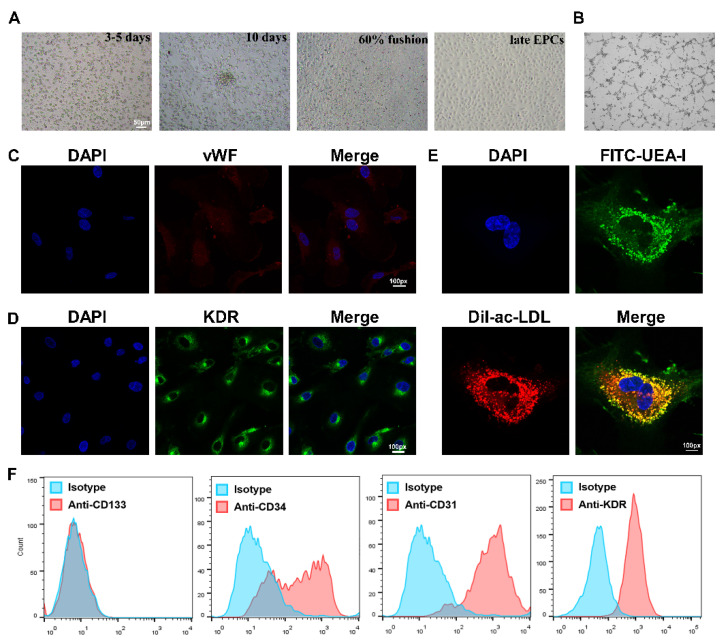
Isolation, culture and identification of EPCs. (**A**) The EPC morphology at different stages was observed under a light microscope, from left to right: the growth at 3–5 days after the cells adhered; the growth at about 10 days; the growth when cells reached more than 60% fusion; the late EPCs after passage. Scale bar, 50 µm. (**B**) The angiogenic function of EPCs was evaluated by tube formation assays. (**C**) Confirmation of the isolation of EPCs via vWF staining (red), where cell nuclei were stained with DAPI (blue). Magnification, ×100. (**D**) Identification of KDR expression (green) in EPCs using immunofluorescence assay. FITC-UEA-I is shown in green, and Dil-Ac-LDL is shown in red. Double-positive cells (yellow), were characterized as EPCs. Magnification, ×100. (**E**) Identification of differentiated EPCs via Dil-Ac-LDL and FITC-UEA-I staining. Magnification, ×100. (**F**) Identification of EPCs expressing CD133, CD34, CD31, and KDR by flow cytometry analysis. EPCs were negative for the stem-cell maker CD133, but positive for the progenitor cell marker CD34, as well as the endothelial cell markers CD31 and KDR. Isotype controls are shown as blue lines.

EPCs were isolated from the PBMCs of healthy subjects and AMI patients, and then cultured in conditional medium according to previous protocol [7]. The classic cobblestone-like characteristic was observed, and EPCs were seen to possess tube formation activity (Figure 1A,B). Cells were identified by immunofluorescence staining, and both KDR and vWF stains were positive (Figure 1C,D).

The colocalization of FITC-UEA-I and Dil-ac-LDL was also observed on the cell surface (Figure 1E). Lastly, the flow cytometry results revealed that the cells were positive for CD34, as well as the endothelium-associated surface antigen markers CD31 and KDR, but the stem-cell surface antigen CD133 was barely expressed (Figure 1F). These results suggest that the isolated cells were late EPCs and could be used in further study.

### 3.2. The Number of EPCs in the Peripheral Blood of AMI Patients Was Higher than That of Healthy Subjects

**Figure 2:**
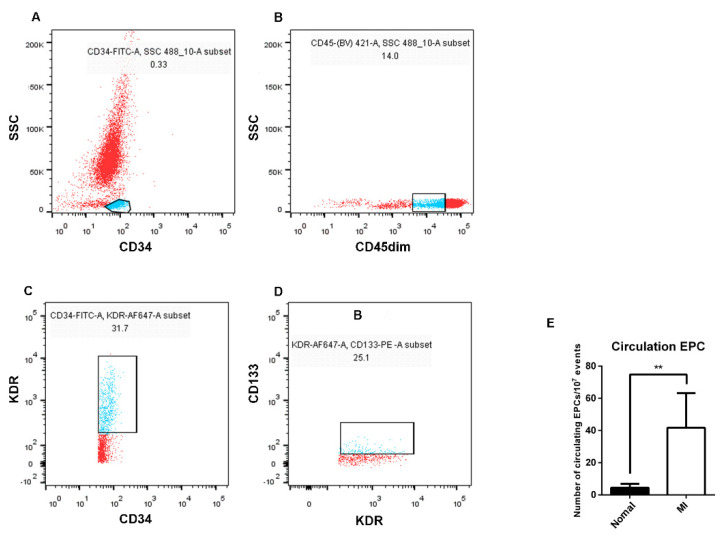
Differences in the number of EPCs in the peripheral blood between AMI patients and healthy subjects by flow cytometry analysis. (**A**) FITC−labeled CD34−positive cell population (blue) was selected from peripheral blood. (**B**) BV421−labeled CD45dim cell population (blue) was selected from the CD34−positive cell population. (**C**) The cell population of Alexa Flour 647−labeled KDR (blue) was selected from the CD45dim-positive cell population. (**D**) The PE−labeled CD133 cell population (blue) was selected from the KDR−positive cell population. (**E**) Statistics and analysis of flow cytometry results (seven in the healthy subjects and six in the AMI patients). Normal: healthy subjects; MI: AMI patients. Data are shown as the mean ± SEM; ** *p* < 0.01.

To understand the main differences of EPCs between AMI patients and healthy subjects, the number of EPCs in the peripheral blood was firstly examined. CD34-positive mononuclear cell populations were isolated, then CD45dim cells were gated from the CD34-positive cell populations, and EPCs were identified by KDR and CD133 staining in turn, counted by the percentage of mutual superposition (Figure 2). The results showed that the number of EPCs in the peripheral blood of AMI patients was higher than that of healthy subjects.

### 3.3. The Migration Ability of EPCs in the Peripheral Blood of AMI Patients Was Stronger That of than Healthy Subjects

**Figure 3:**
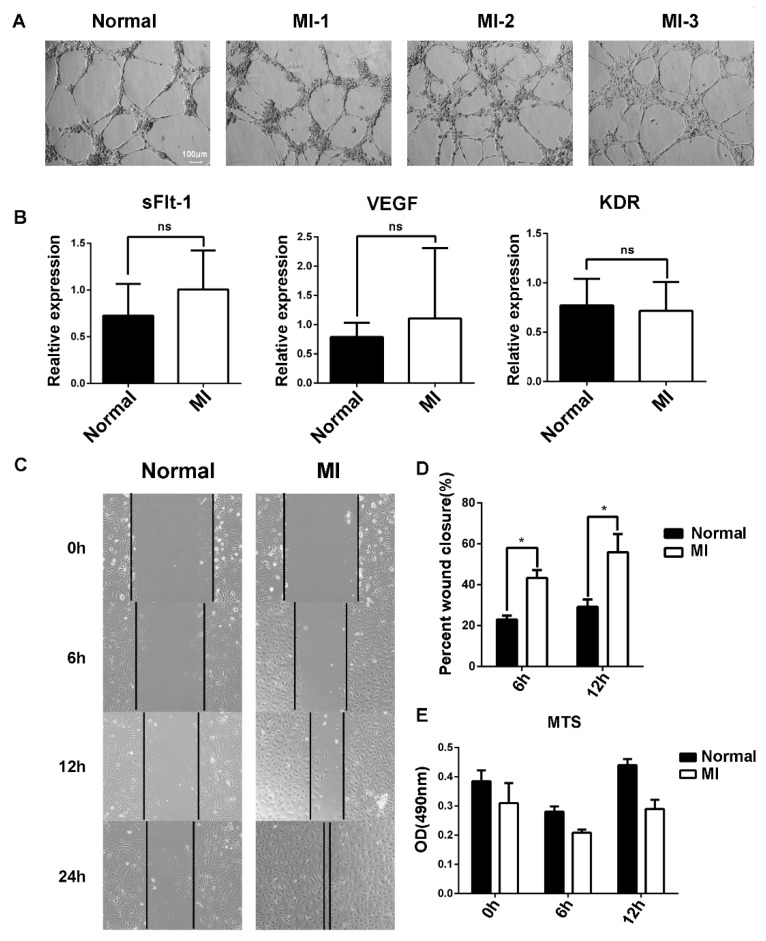
Differences in the function of EPCs between AMI patients and healthy subjects. (**A**) Comparison of angiogenesis of EPCs between healthy subjects and AMI patients. Normal: healthy subjects; MI-1/MI-2/MI-3: AMI patients. (**B**) Expression level of sFlt-1/VEGF/KDR mRNA of EPCs in AMI patients and healthy subjects as determined by RT-PCR. (**C**) EPC migration was examined using the scratch assay, and the scratch images at 0 h, 6 h, 12 h, and 24 h were recorded. (**D**) Statistical analysis of scratch assay. (**E**) Comparison of EPC proliferation ability between AMI patients and healthy subjects according to the MTS assay. Data are shown as the mean ± SEM; * *p* < 0.05, ns, not significant.

The angiogenesis of EPCs was further examined using Matrigel, and no difference was found between AMI patients and healthy subjects (Figure 3A). Vascular endothelial growth factor (VEGF) is crucial for angiogenesis. VEGF binding to its receptor VEGFR2 stimulates key downstream signaling cascades that affect EC angiogenesis. We examined the mRNA expression of VEGF and VEGFR2 by RT-qPCR, and the results showed that there was no difference between AMI patients and healthy subjects (Figure 3B).

It has been reported that soluble tyrosine kinase 1 (sFlt-1) binds to VEGF and blocks the binding of VEGF to VEGFR2, resulting in abnormal blood vessel formation. No difference in sFlt-1 mRNA expression was found (Figure 3B). Then, the migration of EPCs was examined using the scratch method (Figure 3C). Statistical analysis of the scratch images at different timepoints showed that the migration ability of EPCs in AMI patients was stronger than that in healthy subjects (Figure 3D). Moreover, the increased migration ability was probably not due to the cell viability of EPCs between AMI patients and healthy subjects (Figure 3E).

### 3.4. sFlt-1 in the Serum of AMI Patients Inhibited EPC Angiogenesis

**Figure 4:**
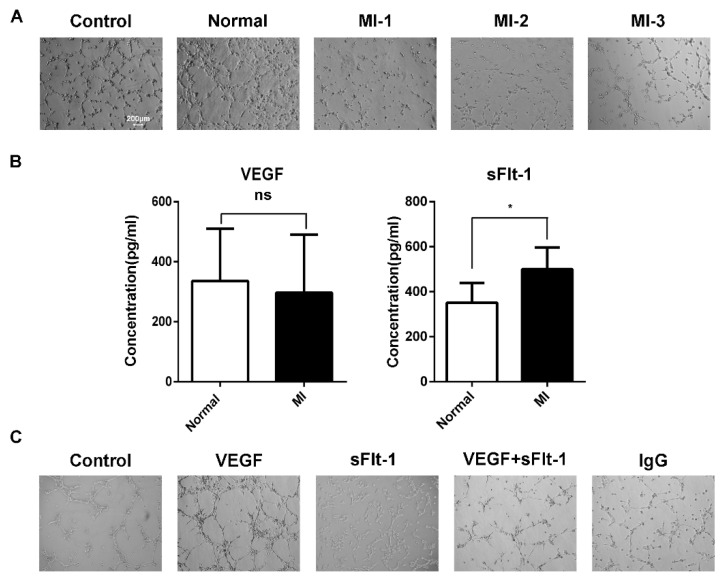
sFlt-1 in the serum from AMI patients inhibits the angiogenesis of EPCs. (**A**) The ability of AMI patients’ serum to inhibit the angiogenesis of EPCs. Control, EPCs cultured in serum-free medium; normal, EPCs cultured in medium containing serum from healthy subjects; MI-1/MI-2/MI-3, EPCs cultured in medium containing serum from different AMI patients. (**B**) ELISA assay of VEGF and sFlt-1 in serum from AMI patients and healthy subjects. Data are shown as the mean ± SEM; * *p* < 0.05, ns, not significant. (**C**) Effect of VEGF and sFlt-1 on the angiogenesis of EPCs.

We found that the increasing number of EPCs in AMI patients seemed contradictory with clinical poor prognoses. Hence, we hypothesized that there might be some inhibitory factors in serum to suppress angiogenesis. Using serum from healthy subjects and AMI patients to stimulate EPCs of healthy subjects, we found that the serum from AMI patients inhibited the tubule formation of EPCs (Figure 4A). When acute myocardial infarction occurs, sudden ischemia occurs, and the body induces a response through angiogenesis and arteriogenesis, thereby improving myocardial perfusion [24]. During the compensatory response, the body secretes various growth factors. The sera of AMI patients and healthy subjects were tested by ELISA, and we found that there was no difference in VEGF between AMI patients and healthy subjects, while the level of sFlt-1 was higher in AMI serum (Figure 4B).

To further confirm whether sFlt-1 is involved in the angiogenesis inhibition of AMI serum, we treated EPCs with VEGF, sFlt-1, VEGF + sFlt-1, and control IgG. The results showed that, compared to VEGF and IgG, sFlt-1 treatment indeed inhibited the angiogenesis ability of EPCs (Figure 4C).

### 3.5. Serum from AMI Patients Promoted the Production of sFlt-1 by EPCs

**Figure 5:**
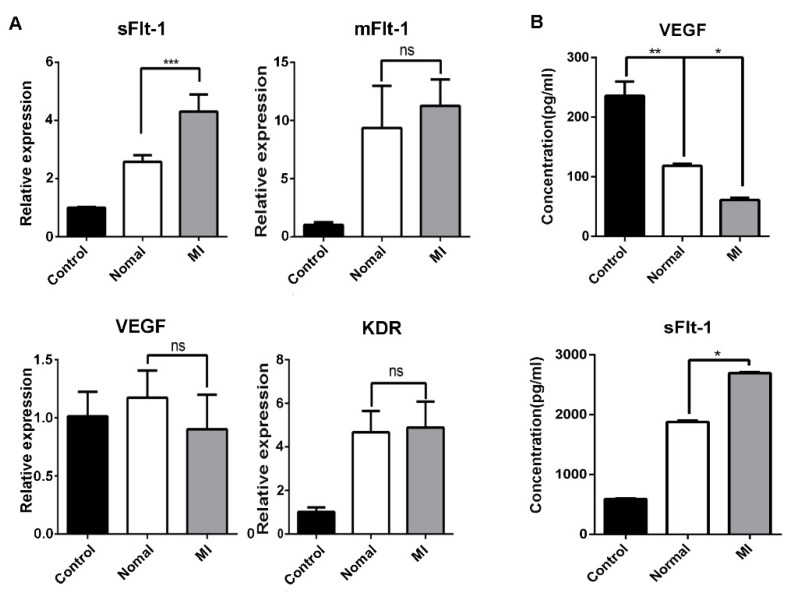
Serum from AMI patients promotes the production of sFlt-1 by EPCs. (**A**) After EPCs were treated with serum from healthy subjects and AMI patients, the mRNA expression level of sFlt-1/mFlt-1/VEGF/KDR was analyzed by RT-PCR. (**B**) ELISA was used to detect the content of VEGF and sFlt-1 in the medium supernatants of EPCs treated with serum from AMI patients and healthy subjects for 4 h. Control, serum-free group; normal, healthy subjects’ serum; MI: AMI patients’ serum. Data are shown as the mean ± SEM; * *p* < 0.05, ** *p* < 0.01, *** *p* < 0.001; ns, not significant.

Endothelial cells represent one of the main sources of sFlt-1. We detected the mRNA expression of sFlt-1, mFlt-1, VEGF, and KDR in EPCs, and we found that only sFlt-1 was increased after challenge by serum from AMI patients (Figure 5A). Similar results were observed by ELISA from the medium supernatants of EPCs after treated by serum from AMI patients or healthy subjects (Figure 5B).

**Figure 6:**
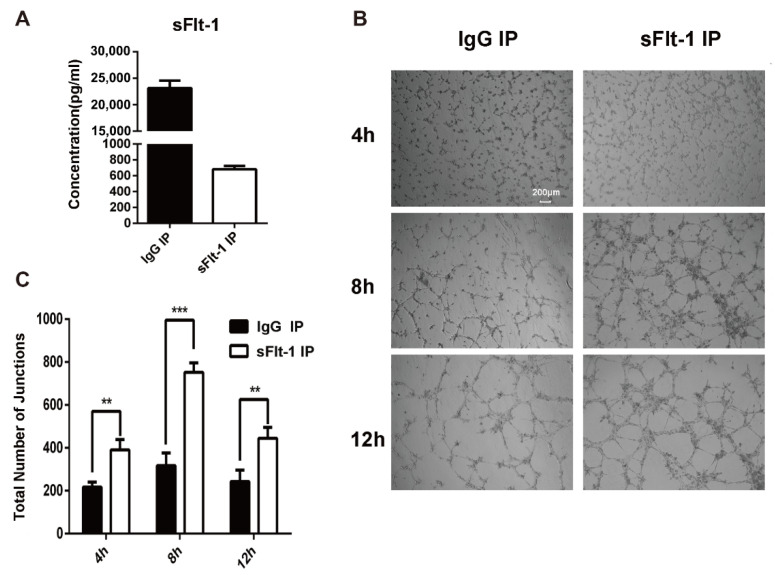
Verification of sFlt-1 in the medium supernatant inhibits the angiogenesis of EPCs. (**A**) sFlt-1 in the medium supernatant after removal by immunoprecipitation was detected by ELISA. (**B**) sFlt-1 was removed from the culture medium supernatant using immunoprecipitation (IP). The medium supernatants with sFlt-1 removed were used to treat EPCs, and the angiogenesis was recorded at 4 h, 8 h and 12 h. (**C**) Statistical analysis of the number of sprouts of tube formation. Data are shown as the mean ± SEM; ** *p* < 0.01, *** *p* < 0.001. sFlt-1 IP: sFlt-1 was removed from the culture medium supernatant using IP. IgG IP: control, sFlt-1 was not removed from the culture medium supernatant using IP.

Then sFlt-1 was removed from the culture medium supernatant using immunoprecipitation (IP). The ELISA result showed that the sFlt-1 was successfully removed from the medium supernatant (Figure 6A). The medium supernatants with no sFlt-1 were used to treat EPCs again, and the angiogenesis was recorded at 4 h, 8 h and 12 h (Figure 6B). Results showed that the removal of sFlt-1 from the medium supernatant removed the ability to inhibit EPC angiogenesis (Figure 6C). Taken together, serum from AMI patients promotes the production of sFlt-1 by EPCs and inhibits the angiogenesis.

### 3.6. Akt and Erk 1/2 Signaling Were Hijacked by sFlt-1 to Inhibit Angiogenesis

**Figure 7:**
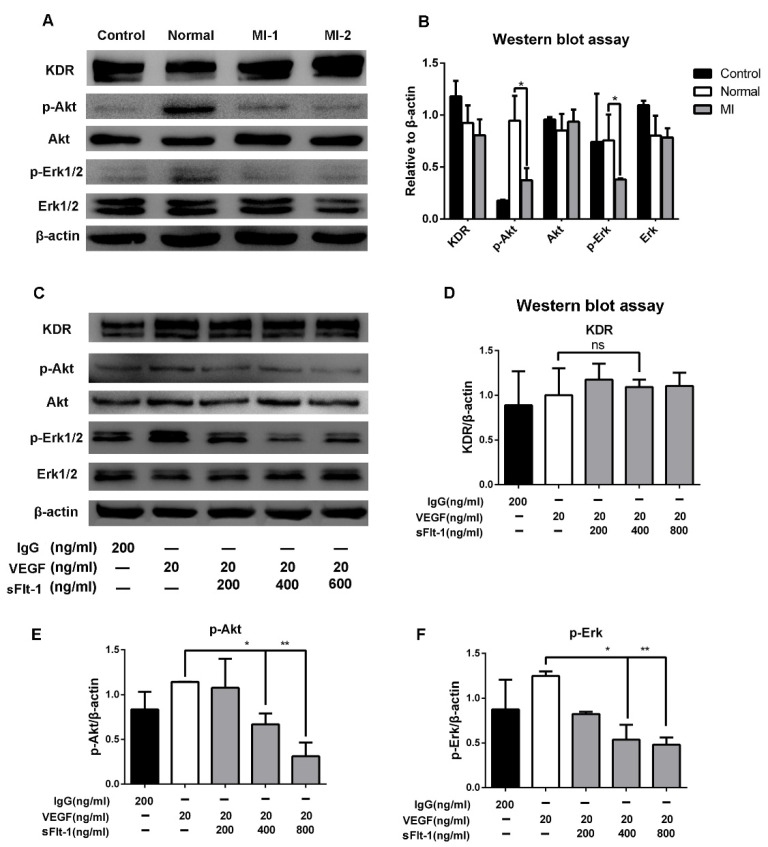
sFlt-1 in the serum from AMI patients inhibits the Akt and Erk 1/2 signaling pathways in EPCs. (**A**) After EPCs were treated with serum from healthy subjects and AMI patients, KDR, p-Akt/Akt, p-Erk/Erk, and β-actin protein expression levels were determined by Western blot. The uncropped western blot figures were presented in Figure S1. (**B**) The gray intensity of protein expression level was determined by ImageJ software. (**C**) The protein expression levels of KDR, p-Akt/Akt, p-Erk/Erk, and β-actin after EPCs were treated with VEGF and sFlt-1, analyzed by Western blot. IgG was used as a negative control; β-actin was used as a loading control. The protein expression levels of KDR (**D**), p-Akt (**E**), and p-Erk (**F**) were analyzed in gray intensity by ImageJ software. Data are shown as the mean ± SEM; * *p* < 0.05, ** *p* < 0.01, ns, not significant. The uncropped western blot figures were presented in Figure S1.

To study the underlying mechanism of sFlt-1 in EPC angiogenesis inhibition, we focused on Akt and Erk signaling, which play a pivotal role in angiogenesis. We found that serum from AMI patients significantly downregulated p-Akt and p-Erk 1/2, but not KDR (Figure 7A,B). Furtherly, the expression levels of KDR, Akt, and Erk 1/2 in EPCs were detected after VEGF and sFlt-1 treatments. The results showed that the upregulation of p-Akt and p-Erk 1/2 by VEGF was reversed when sFlt-1 was present (Figure 7C–F). These results suggest that sFlt-1 from AMI patients serum inhibits angiogenesis via Akt and Erk 1/2 signaling.

## 4. Discussion

Acute myocardial infarction (AMI) remains the main cause of mortality worldwide. After AMI, the recovery of collateral circulation and microvessels requires the participation of EPCs. In this study, we found that the sFlt-1 level in the serum of AMI patients was significantly higher than that of healthy subjects. We also found EPCs to be an important source of sFlt-1, which inhibits their angiogenesis, forming a positive feedback loop.

Our results demonstrated that the number of EPCs in the peripheral blood of AMI patients was higher than that of healthy subjects (Figure 2), which is consistent with the findings of previous studies [8,25]. AMI is considered the strongest stimulus for EPC mobilization [26]. Inflammatory cytokines are released from ischemic tissues, and EPCs are mobilized from bone marrow to the peripheral circulation in response to myocardial ischemia [27]. The elevated circulating EPCs likely contribute to neovascularization. Most AMI patients are accompanied by other diseases, such as hypertension, diabetes, and atherosclerosis. It has been reported that the number and function of EPCs are reduced among participants with hypertension when compared to the healthy population [28,29]. Most AMI patients recruited in our study had hypertension. Since hypertensive patients were at different stages, there may have been an effect on the number of EPCs in the peripheral circulation. However, the increased number of EPCs in the peripheral blood of AMI patients was observed despite a higher incidence of hypertension. Our further understanding of hypertension and EPCs also requires detecting the number of EPCs in the peripheral circulation of hypertensive patients at different stages.

Migration and angiogenesis abilities represent the key characteristics of EPCs. No significant difference in the angiogenesis of EPCs between AMI patients and healthy subjects was observed in this study, while the migration ability of EPCs in AMI patients was stronger than that in healthy subjects (Figure 3). Accordingly, it is reasonable to predict that EPCs were mobilized after AMI and performed a function as a lifeguard [30]. However, this seems paradoxical to the poor prognosis of AMI patients in clinic. Therefore, there must be some other inhibitory factors involved.

It has been well demonstrated that proangiogenic factors such as VEGF and angiogenin are increased when the body is in ischemia or hypoxia [31,32]. However, it has also been shown that serum from cardiovascular patients contains factors that inhibit angiogenesis, such as sFlt-1, TSP-1 (platelet-derived factor), and VEGF-165b (vascular endothelial growth factor-165b) when myocardial infarction occurs [33,34]. Willibald et al. reported that the serum level of sFlt-1 was significantly increased during ongoing AMI [35]. sFlt-1 was reported to be a useful biomarker for AMI in addition to cardiac troponin, indicating that sFlt-1 might play an important role in AMI patients [36]. sFlt-1 can inhibit angiogenesis through VEGF/KDR [37,38]; however, there are few studies on sFlt-1 and EPCs in the peripheral blood of AMI patients.

In this study, we found the sFlt-1 level in AMI patients to be obviously higher than that of healthy subjects. Removing sFlt-1 using immunoprecipitation from the culture medium supernatant reversed the angiogenesis inhibition phenotype, suggesting that EPCs are one of the main sources of sFlt-1 in serum. This is consistent with previous studies verifying that the secretion of sFlt-1 mainly originated from ECs and PBMCs [22,39]. The secretion mechanism of sFlt-1 is related to hypoxia, and the nuclear factor of activated T cells (NFTA) promotes the secretion of sFlt-1 under hypoxic conditions [40].

Phosphatidylinositol-3-kinase (PI3K)/Akt and Erk signaling are involved in the functional regulation of EPCs, such as cell proliferation and migration in angiogenesis. Serum from AMI patients and sFlt-1 significantly inhibited the expression of p-Akt and p-Erk according to Western blot (Figure 7). However, whether the serum contains other inhibitory factors, such as exosomes, needs to be further explored.

## 5. Conclusions

In this study, EPCs were isolated from human peripheral blood and identified as late-stage EPCs by flow cytometry, immunofluorescence, and blood vessel formation assay. We found that EPCs in the peripheral blood of AMI patients were greater in number than those of healthy subjects, and the migration ability of EPCs from AMI patients was stronger, but there was no difference in the angiogenesis and expression levels of sFlt-1/VEGF/KDR. Further studies revealed that serum from AMI patients inhibited the angiogenesis of EPCs and promoted the secretion of sFlt-1 from EPCs. sFlt-1 in the serum from AMI patients was shown to inhibit the angiogenesis of EPCs by inhibiting the Akt and Erk signaling pathways.

## Figures and Tables

**Table 1:** RT-PCR primers of target genes.

Gene Name	Sequence	
	Forward	Reverse
β-actin	CCACCATGTACCCTGGCATT	CGGACTCGTCATACTCCTGC
VEGF	GGGCCTCCGAAACCATGAACTT	AGGGGCACACAGGATGGCTTGA
sFlt-1	GCGCATGGCAATAATAGAAGGAAA	CCTTTTTGTTGCAGTGCTCACCTC
mFlt-1	GCCCGGGAGAGACTTAAACTGG	GGCCTGGCTCCATTTTTTCTTTC
KDR	CAACAAAGTCGGGAGAGGAG	ATGACGATGGACAAGTAGCC

## Data Availability

Available data are presented in the manuscript and reference list.

## Supplementary Materials

The following supporting information can be downloaded at: https://www.mdpi.com/article/10.3390/biology11081194/s1, Table S1: Patient demographic and clinical characteristics. The uncropped western blot figures were presented in Figure S1.
