# Supplementary material for: Soluble Flt-1 in AMI Patients Serum Inhibits Angiogenesis of Endothelial Progenitor Cells by Suppressing Akt and Erk’s Activity
Source: Biology (Basel). 2022 Aug 9;11(8):1194. doi: 10.3390/biology11081194 (PMC9404789; doi:10.3390/biology11081194)
Supplement: Supplementary file 1 [file biology-11-01194-s001.zip › biology-1793376-supplementary.pdf]

Table S1. Patient demographic and clinical characteristics.

Patient No.	Age	Gender	ischemic time	DM	HT	Smoking
1	69	F	4h	No	No	No
2	77	F	6h	No	Yes	No
3	37	M	4.5h	No	Yes	No
4	61	M	3.5h	No	No	No
5	69	M	3h	Yes	Yes	Yes
6	66	F	3.5h	No	Yes	No
7	69	M	2h	No	Yes	No

F—female; M—male; DM—diabetes mellitus; HT—hypertension

Figure S1

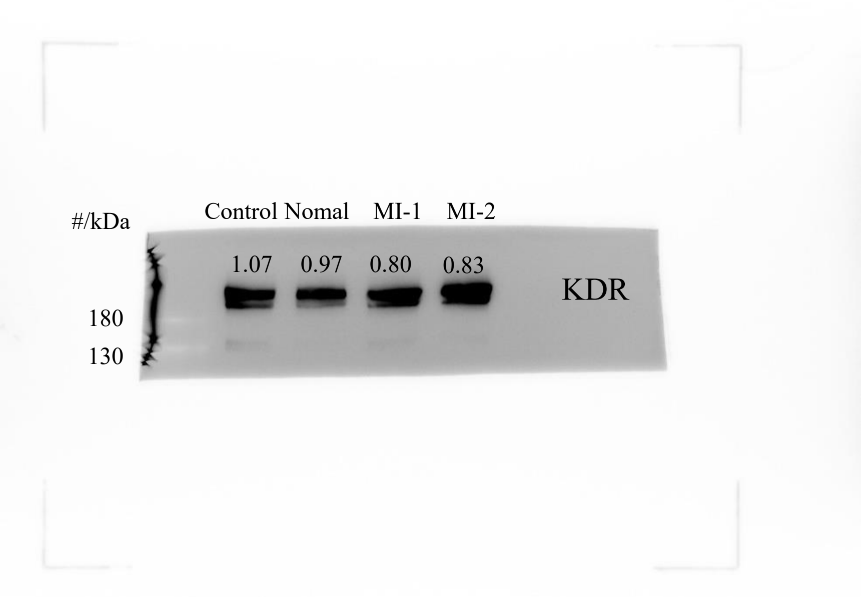

Western blot membrane of KDR (~210 kDa) protein detected with anti-KDR antibody (Rabbit mAb; 2479; 1:1000; CST). Proteins were denatured with SDS sample buffer at 100°C for 10 minutes and then separated by SDS-PAGE gels and transferred onto NC membranes. After blocked with TBST containing 5% BSA, membranes were incubated with KDR antibodies overnight at 4°C. Then, the membranes were incubated with the appropriate secondary antibodies coupled to HRP for 2 hours at room temperature. The protein bands were detected with Pierce™ ECL Western Blotting Substrate and scanned by an automatic chemiluminescence imaging system (Tanon, Shanghai, China). The number above the band represents the intensity ratio of the target protein and the corresponding β-actin. #Weight marker (molecular weight in kDa): Thermo Scientific™/PageRuler™ Prestained Protein Ladder, 10 to 180 kDa; catalogue number: 26616. Blot images, prior to the densitometry readings, were converted to grayscale with ImageJ (ImageJ v.1.49, National Institutes of Health, Maryland, USA) as follows: Image -> Type –> 8 bit, next: Image -> Adjust–> Brightness/Contrast –> Auto. WL.

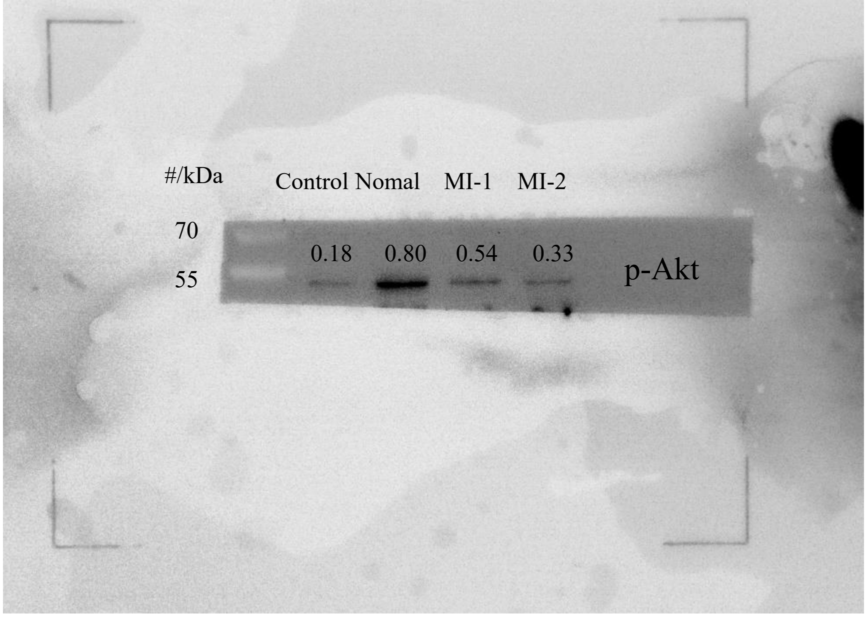

Western blot membrane of p-Akt (~60 kDa) protein detected with anti-p-Akt antibody (Phospho-Akt (Ser473) (D9E) XP® Rabbit mAb; 4060; 1:1000; CST). Proteins were denatured with SDS sample buffer at 100°C for 10 minutes and then separated by SDS-PAGE gels and transferred onto NC membranes. After blocked with TBST containing 5% BSA, membranes were incubated with KDR antibodies overnight at 4°C. Then, the membranes were incubated with the appropriate secondary antibodies coupled to HRP for 2 hours at room temperature. The protein bands were detected with Pierce™ ECL Western Blotting Substrate and scanned by an automatic chemiluminescence imaging system (Tanon, Shanghai, China). The number above the band represents the intensity ratio of the target protein and the corresponding β-actin. #Weight marker (molecular weight in kDa): Thermo Scientific™/PageRuler™ Prestained Protein Ladder, 10 to 180 kDa; catalogue number: 26616. Blot images, prior to the densitometry readings, were converted to grayscale with ImageJ (ImageJ v.1.49, National Institutes of Health, Maryland, USA) as follows: Image -> Type –> 8 bit, next: Image -> Adjust–> Brightness/Contrast –> Auto. WL.

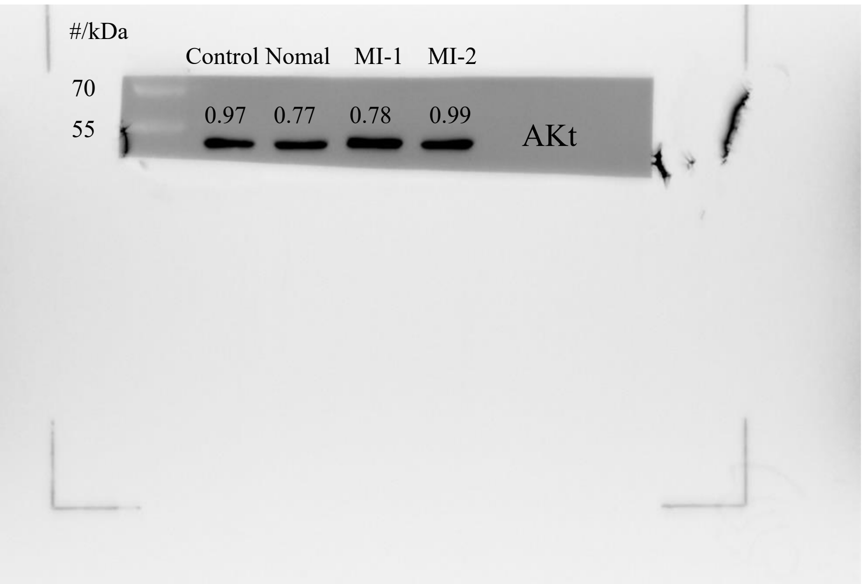

Western blot membrane of Akt (~60 kDa) protein detected with anti-Akt antibody (Akt (pan) (11E7) Rabbit mAb; 4685; 1:1000; CST). Proteins were denatured with SDS sample buffer at 100°C for 10 minutes and then separated by SDS-PAGE gels and transferred onto NC membranes. After blocked with TBST containing 5% BSA, membranes were incubated with KDR antibodies overnight at 4°C. Then, the membranes were incubated with the appropriate secondary antibodies coupled to HRP for 2 hours at room temperature. The protein bands were detected with Pierce™ ECL Western Blotting Substrate and scanned by an automatic chemiluminescence imaging system (Tanon, Shanghai, China). The number above the band represents the intensity ratio of the target protein and the corresponding β-actin. #Weight marker (molecular weight in kDa): Thermo Scientific™/PageRuler™ Prestained Protein Ladder, 10 to 180 kDa; catalogue number: 26616. Blot images, prior to the densitometry readings, were converted to grayscale with ImageJ (ImageJ v.1.49, National Institutes of Health, Maryland, USA) as follows: Image -> Type –> 8 bit, next: Image -> Adjust–> Brightness/Contrast –> Auto. WL.

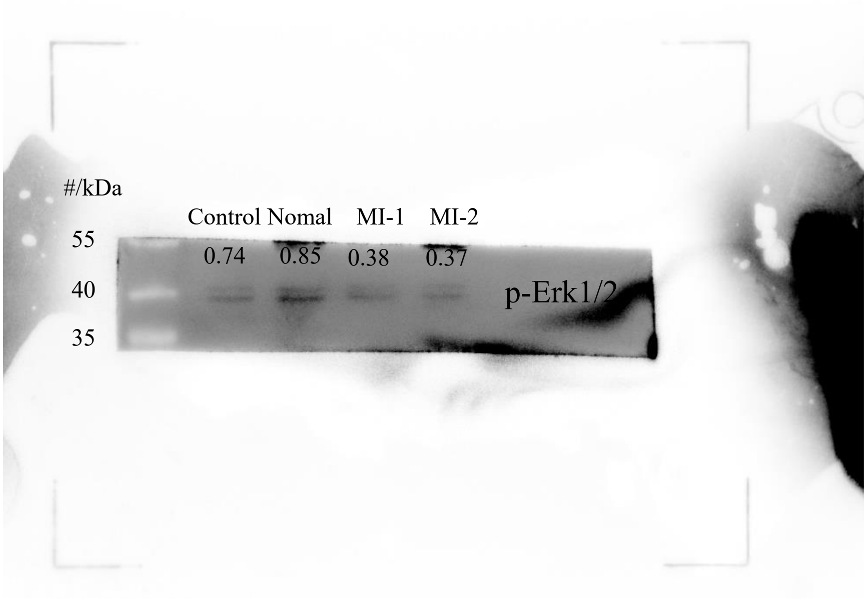

Western blot membrane of p-Erk1/2 (~44/42 kDa) protein detected with anti-p-Erk1/2 antibody (Phospho-p44/42 MAPK (Erk1/2) (Thr202/Tyr204) (E10) Mouse mAb; 9106; 1:1000; CST). Proteins were denatured with SDS sample buffer at 100°C for 10 minutes and then separated by SDS-PAGE gels and transferred onto NC membranes. After blocked with TBST containing 5% BSA, membranes were incubated with KDR antibodies overnight at 4°C. Then, the membranes were incubated with the appropriate secondary antibodies coupled to HRP for 2 hours at room temperature. The protein bands were detected with Pierce™ ECL Western Blotting Substrate and scanned by an automatic chemiluminescence imaging system (Tanon, Shanghai, China). The number above the band represents the intensity ratio of the target protein and the corresponding β-actin. #Weight marker (molecular weight in kDa): Thermo Scientific™/PageRuler™ Prestained Protein Ladder, 10 to 180 kDa; catalogue number: 26616. Blot images, prior to the densitometry readings, were converted to grayscale with ImageJ (ImageJ v.1.49, National Institutes of Health, Maryland, USA) as follows: Image -> Type –> 8 bit, next: Image -> Adjust–> Brightness/Contrast –> Auto. WL.

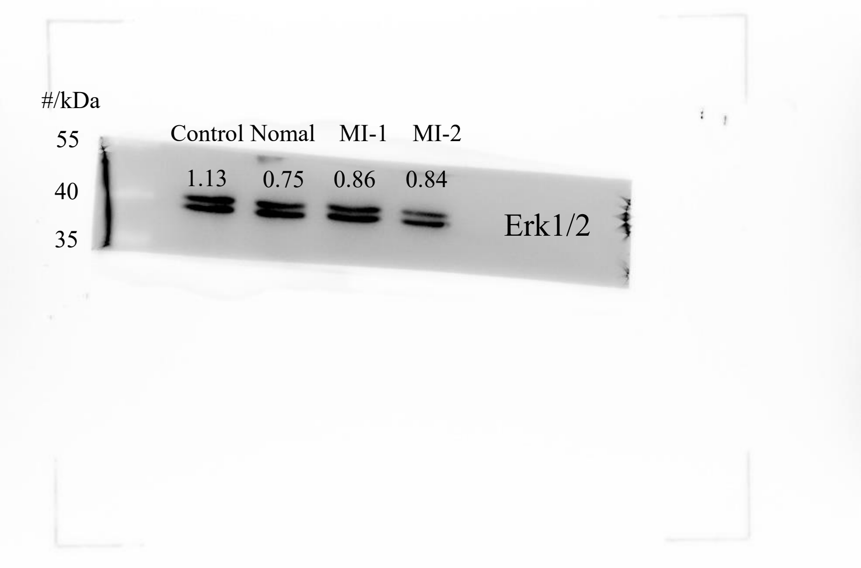

Western blot membrane of Erk1/2 (~44/42 kDa) protein detected with block Erk1/2 peptide (p44/42 MAPK (Erk1/2) Blocking Peptide; 4695; 1:1000; CST). Proteins were denatured with SDS sample buffer at 100°C for 10 minutes and then separated by SDS-PAGE gels and transferred onto NC membranes. After blocked with TBST containing 5% BSA, membranes were incubated with KDR antibodies overnight at 4°C. Then, the membranes were incubated with the appropriate secondary antibodies coupled to HRP for 2 hours at room temperature. The protein bands were detected with Pierce™ ECL Western Blotting Substrate and scanned by an automatic chemiluminescence imaging system (Tanon, Shanghai, China). The number above the band represents the intensity ratio of the target protein and the corresponding β-actin. #Weight marker (molecular weight in kDa): Thermo Scientific™/PageRuler™ Prestained Protein Ladder, 10 to 180 kDa; catalogue number: 26616. Blot images, prior to the densitometry readings, were converted to grayscale with ImageJ (ImageJ v.1.49, National Institutes of Health, Maryland, USA) as follows: Image -> Type –> 8 bit, next: Image -> Adjust–> Brightness/Contrast –> Auto. WL.

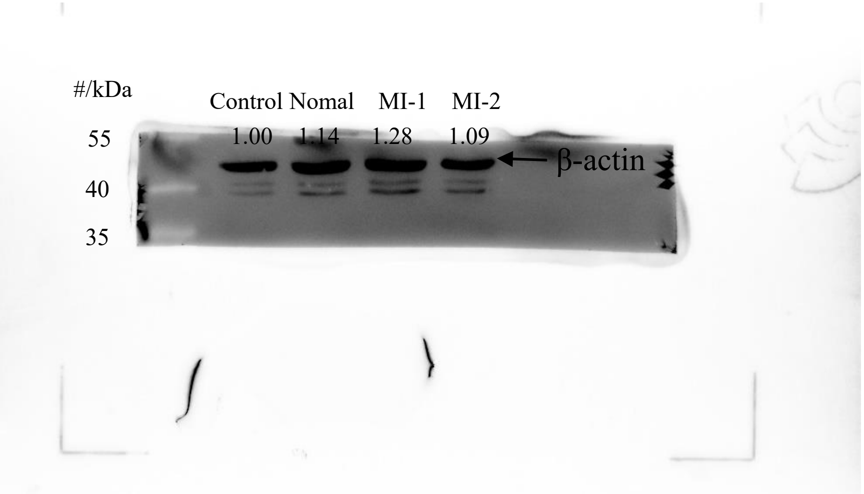

Western blot membrane of β-actin (~42 kDa) protein detected with anti-β-actin antibody (A1978; 1:1000; Sigma-Aldrich). Proteins were denatured with SDS sample buffer at 100°C for 10 minutes and then separated by SDS-PAGE gels and transferred onto NC membranes. After blocked with TBST containing 5% BSA, membranes were incubated with KDR antibodies overnight at 4°C. Then, the membranes were incubated with the appropriate secondary antibodies coupled to HRP for 2 hours at room temperature. The protein bands were detected with Pierce™ ECL Western Blotting Substrate and scanned by an automatic chemiluminescence imaging system (Tanon, Shanghai, China). The number above the band represents intensity ratio relative to the first β-actin. #Weight marker (molecular weight in kDa): Thermo Scientific™/PageRuler™ Prestained Protein Ladder, 10 to 180 kDa; catalogue number: 26616. Blot images, prior to the densitometry readings, were converted to grayscale with ImageJ (ImageJ v.1.49, National Institutes of Health, Maryland, USA) as follows: Image -> Type –> 8 bit, next: Image -> Adjust–> Brightness/Contrast –> Auto. WL.

Fig 7

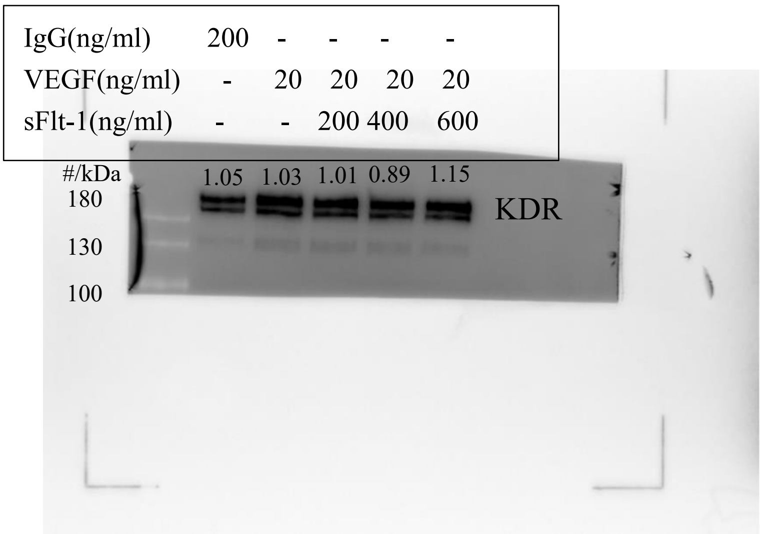

Western blot membrane of KDR (~210 kDa) protein detected with anti-KDR antibody (Rabbit mAb; 2479; 1:1000; CST). Proteins were denatured with SDS sample buffer at 100°C for 10 minutes and then separated by SDS-PAGE gels and transferred onto NC membranes. After blocked with TBST containing 5% BSA, membranes were incubated with KDR antibodies overnight at 4°C. Then, the membranes were incubated with the appropriate secondary antibodies coupled to HRP for 2 hours at room temperature. The protein bands were detected with Pierce™ ECL Western Blotting Substrate and scanned by an automatic chemiluminescence imaging system (Tanon, Shanghai, China). The number above the band represents the intensity ratio of the target protein and the corresponding β-actin. #Weight marker (molecular weight in kDa): Thermo Scientific™/PageRuler™ Prestained Protein Ladder, 10 to 180 kDa; catalogue number: 26616. Blot images, prior to the densitometry readings, were converted to grayscale with ImageJ (ImageJ v.1.49, National Institutes of Health, Maryland, USA) as follows: Image -> Type –> 8 bit, next: Image -> Adjust–> Brightness/Contrast –> Auto. WL.

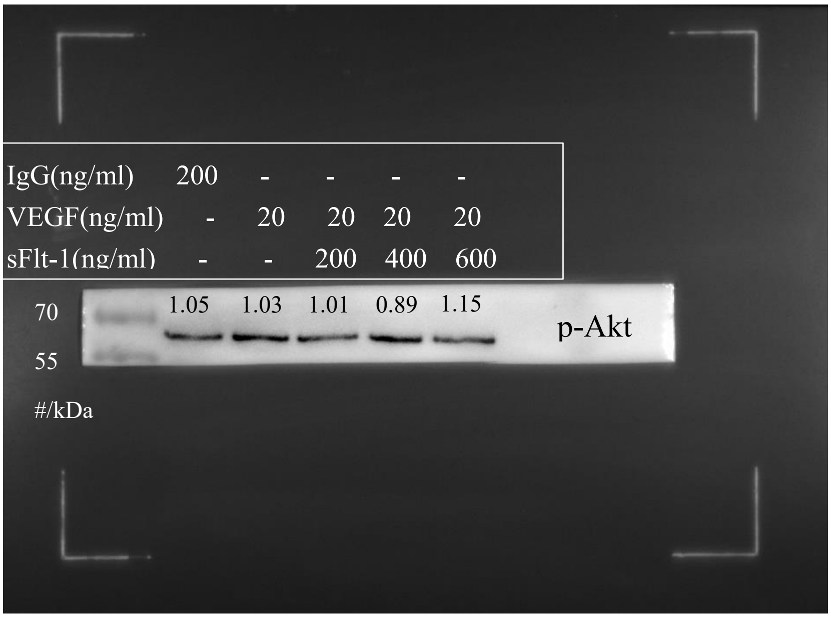

Western blot membrane of p-Akt (~60 kDa) protein detected with anti-p-Akt antibody (Phospho-Akt (Ser473) (D9E) XP® Rabbit mAb; 4060; 1:1000; CST). Proteins were denatured with SDS sample buffer at 100°C for 10 minutes and then separated by SDS-PAGE gels and transferred onto NC membranes. After blocked with TBST containing 5% BSA, membranes were incubated with KDR antibodies overnight at 4°C. Then, the membranes were incubated with the appropriate secondary antibodies coupled to HRP for 2 hours at room temperature. The protein bands were detected with Pierce™ ECL Western Blotting Substrate and scanned by an automatic chemiluminescence imaging system (Tanon, Shanghai, China). The number above the band represents the intensity ratio of the target protein and the corresponding β-actin. #Weight marker (molecular weight in kDa): Thermo Scientific™/PageRuler™ Prestained Protein Ladder, 10 to 180 kDa; catalogue number: 26616. Blot images, prior to the densitometry readings, were converted to grayscale with ImageJ (ImageJ v.1.49, National Institutes of Health, Maryland, USA) as follows: Image -> Type –> 8 bit, next: Image -> Adjust–> Brightness/Contrast –> Auto. WL.

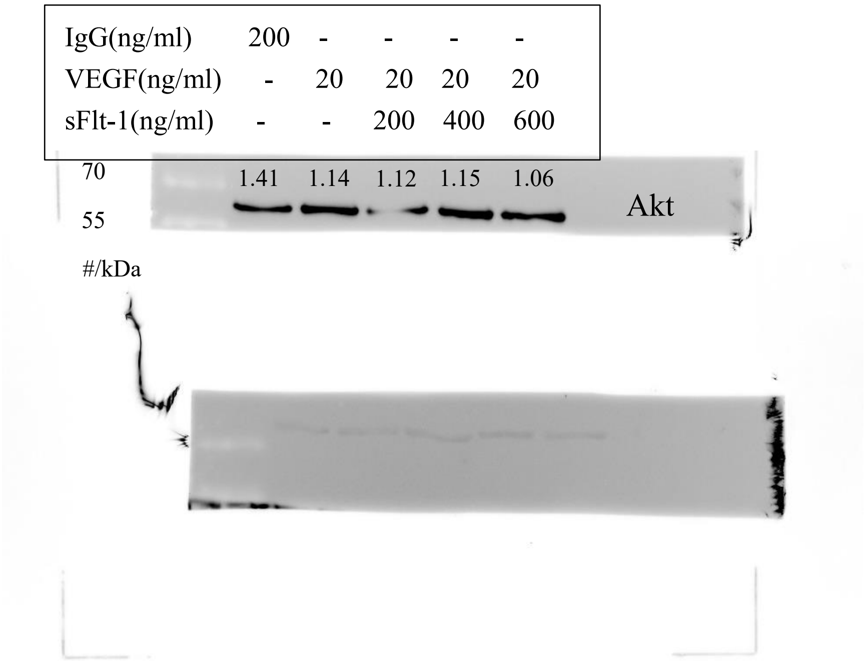

Western blot membrane of Akt (~60 kDa) protein detected with anti-KDR antibody (Akt (pan) (11E7) Rabbit mAb; 4685; 1:1000; CST). Proteins were denatured with SDS sample buffer at 100°C for 10 minutes and then separated by SDS-PAGE gels and transferred onto NC membranes. After blocked with TBST containing 5% BSA, membranes were incubated with KDR antibodies overnight at 4°C. Then, the membranes were incubated with the appropriate secondary antibodies coupled to HRP for 2 hours at room temperature. The protein bands were detected with Pierce™ ECL Western Blotting Substrate and scanned by an automatic chemiluminescence imaging system (Tanon, Shanghai, China). The number above the band represents the intensity ratio of the target protein and the corresponding β-actin. #Weight marker (molecular weight in kDa): Thermo Scientific™/PageRuler™ Prestained Protein Ladder, 10 to 180 kDa; catalogue number: 26616. Blot images, prior to the densitometry readings, were converted to grayscale with ImageJ (ImageJ v.1.49, National Institutes of Health, Maryland, USA) as follows: Image -> Type –> 8 bit, next: Image -> Adjust–> Brightness/Contrast –> Auto. WL.

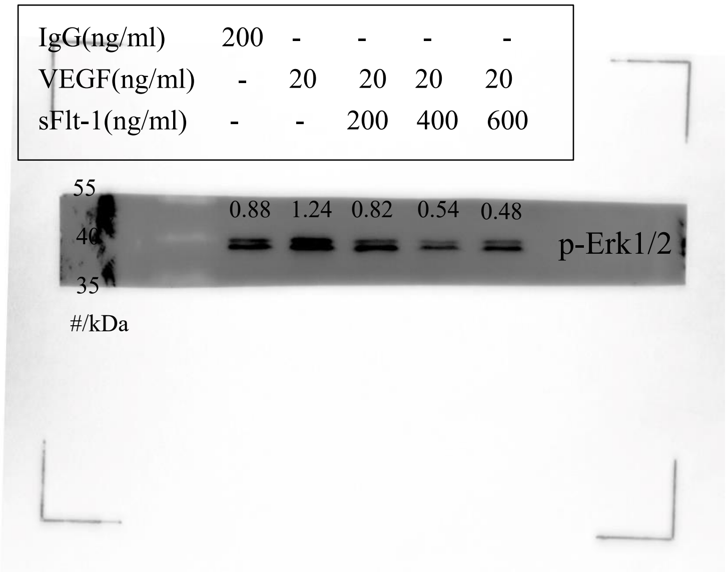

Western blot membrane of p-Erk1/2 (~44/42 kDa) protein detected with block p-Erk1/2 antibody (Phospho-p44/42 MAPK (Erk1/2) (Thr202/Tyr204) (E10) Mouse mAb; 9106; 1:1000; CST). Proteins were denatured with SDS sample buffer at 100°C for 10 minutes and then separated by SDS-PAGE gels and transferred onto NC membranes. After blocked with TBST containing 5% BSA, membranes were incubated with KDR antibodies overnight at 4°C. Then, the membranes were incubated with the appropriate secondary antibodies coupled to HRP for 2 hours at room temperature. The protein bands were detected with Pierce™ ECL Western Blotting Substrate and scanned by an automatic chemiluminescence imaging system (Tanon, Shanghai, China). The number above the band represents the intensity ratio of the target protein and the corresponding β-actin. #Weight marker (molecular weight in kDa): Thermo Scientific™/PageRuler™ Prestained Protein Ladder, 10 to 180 kDa; catalogue number: 26616. Blot images, prior to the densitometry readings, were converted to grayscale with ImageJ (ImageJ v.1.49, National Institutes of Health, Maryland, USA) as follows: Image -> Type –> 8 bit, next: Image -> Adjust–> Brightness/Contrast –> Auto. WL.

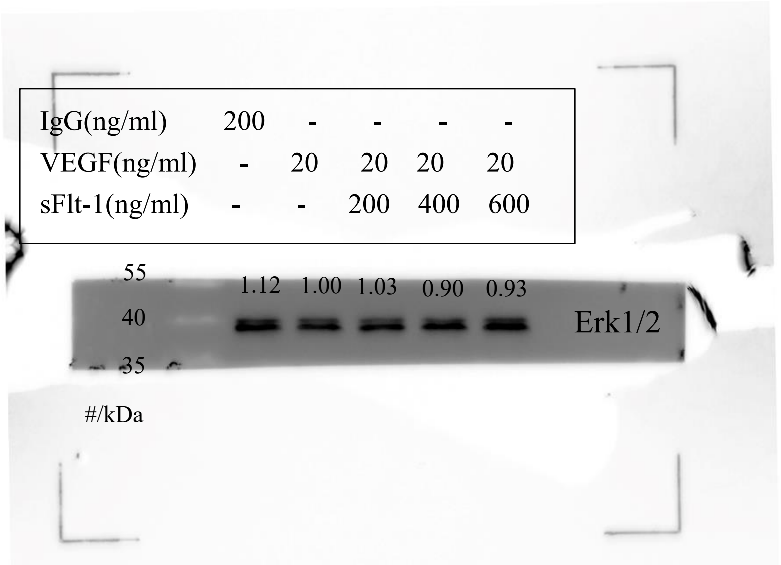

Western blot membrane of Erk1/2 (~44/42 kDa) protein detected with block Erk1/2 peptide (p44/42 MAPK (Erk1/2) Blocking Peptide; 4695; 1:1000; CST). Proteins were denatured with SDS sample buffer at 100°C for 10 minutes and then separated by SDS-PAGE gels and transferred onto NC membranes. After blocked with TBST containing 5% BSA, membranes were incubated with KDR antibodies overnight at 4°C. Then, the membranes were incubated with the appropriate secondary antibodies coupled to HRP for 2 hours at room temperature. The protein bands were detected with Pierce™ ECL Western Blotting Substrate and scanned by an automatic chemiluminescence imaging system (Tanon, Shanghai, China). The number above the band represents the intensity ratio of the target protein and the corresponding β-actin. #Weight marker (molecular weight in kDa): Thermo Scientific™/PageRuler™ Prestained Protein Ladder, 10 to 180 kDa; catalogue number: 26616. Blot images, prior to the densitometry readings, were converted to grayscale with ImageJ (ImageJ v.1.49, National Institutes of Health, Maryland, USA) as follows: Image -> Type –> 8 bit, next: Image -> Adjust–> Brightness/Contrast –> Auto. WL.

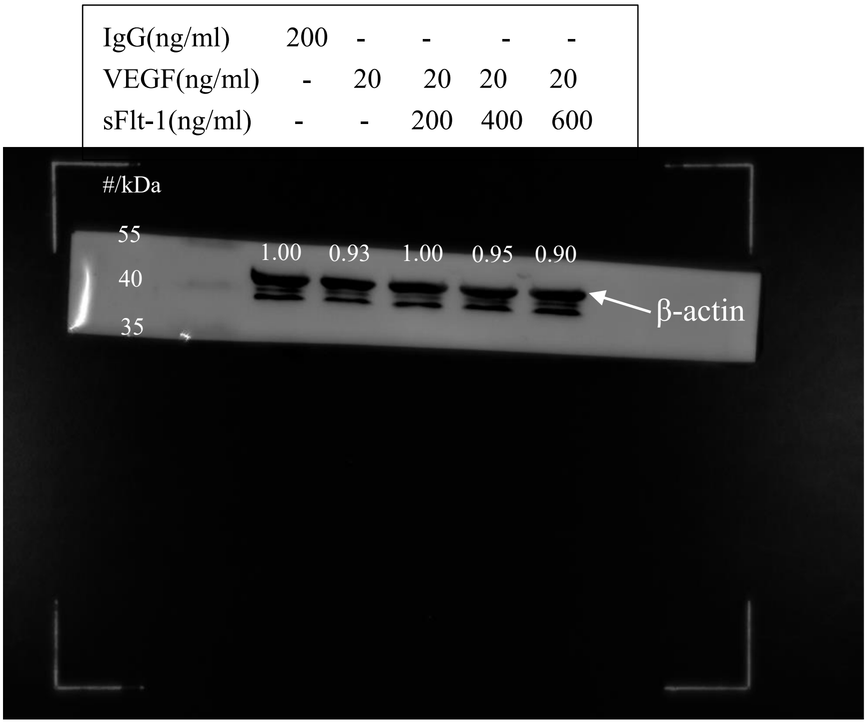

Western blot membrane of β-actin (~42 kDa) protein detected with anti-β-actin antibody (A1978; 1:1000; Sigma-Aldrich). Proteins were denatured with SDS sample buffer at 100°C for 10 minutes and then separated by SDS-PAGE gels and transferred onto NC membranes. After blocked with TBST containing 5% BSA, membranes were incubated with KDR antibodies overnight at 4°C. Then, the membranes were incubated with the appropriate secondary antibodies coupled to HRP for 2 hours at room temperature. The protein bands were detected with Pierce™ ECL Western Blotting Substrate and scanned by an automatic chemiluminescence imaging system (Tanon, Shanghai, China). The number above the band represents intensity ratio relative to the first β-actin. #Weight marker (molecular weight in kDa): Thermo Scientific™/PageRuler™ Prestained Protein Ladder, 10 to 180 kDa; catalogue number: 26616. Blot images, prior to the densitometry readings, were converted to grayscale with ImageJ (ImageJ v.1.49, National Institutes of Health, Maryland, USA) as follows: Image -> Type –> 8 bit, next: Image -> Adjust–> Brightness/Contrast –> Auto. WL.
